# A perspective from the European Network for Bipolar and Emotion Regulation on research and intervention for emotion dysregulation in bipolar disorder: challenges and lessons learned

**DOI:** 10.3389/fpsyt.2024.1518106

**Published:** 2025-02-13

**Authors:** Julieta Azevedo, Alyson Dodd, Luisa Weiner, Katia M’Bailara, Ana M. Pinto, Sébastien Weibel, Caroline Lawlor, Manja Koenders, Kim Wright

**Affiliations:** ^1^ Mood Disorders Centre, Department of Psychology, College of Life and Environmental Sciences, University of Exeter, Exeter, United Kingdom; ^2^ Center for Research of the Center for Cognitive Intervention and Studies, Faculty of Psychology and Educational Sciences, University of Coimbra, Coimbra, Portugal; ^3^ Department of Psychology, Northumbria University, Newcastle, United Kingdom; ^4^ Laboratoire de Psychologie des Cognitions, University of Strasbourg, Strasbourg, France; ^5^ Psychiatry Department, University Hospital of Strasbourg, Strasbourg, France; ^6^ EA4139 Laboratoire de Psychologie, Université de Bordeaux, Bordeaux, France; ^7^ UMRS1329 Strasbourg Translational Neuroscience and Psychiatry, University of Strasbourg, Strasbourg, France; ^8^ North Lambeth Focused Support Team, South London and Maudsley NHS Foundation Trust, London, United Kingdom; ^9^ Department of Psychology, King’s College London, Institute of Psychiatry, Psychology and Neuroscience, London, United Kingdom; ^10^ Faculty of Social Sciences, Leiden University, Leiden, Netherlands

**Keywords:** bipolar disorder, emotion regulation, bipolar depression, mania, emotion dysregulation

## Abstract

Bipolar disorder (BD) and emotion dysregulation present substantial challenges for individuals and healthcare providers. Although pharmacological treatments remain the primary approach, psychosocial interventions show promise in addressing sub-threshold symptoms and deepening understanding of mood and emotion dysregulation mechanisms. The European Network for Bipolar Emotion Regulation (ENBER) aims to close the gap between research and clinical practice by offering practical insights for clinicians while contributing to scientific discourse on BD and emotion regulation (ER). This perspective paper identifies key questions for the field, suggesting directions for future research and highlighting promising interventions, such as Dialectical Behaviour Therapy (DBT), which have shown potential to reduce emotion dysregulation and improve personal recovery in BD. Future research should explore the flexibility and context-appropriateness of ER strategies, considering how current mood states significantly impact these dynamics. The commentary advocates for personalised treatment approaches that address individual differences in symptoms and ER capabilities, recommending innovative methodologies to better understand and apply ER in BD.Incorporating patient perspectives into research design is also a necessary focus for future research, having the potential to improve recovery and quality of life for individuals with BD.

## Introduction

Recent guidelines from the National Institute for Health and Care Excellence (NICE) and other international organisations (Canadian Network for Mood and Anxiety Treatments- CANMAT, 1; Agency for Healthcare Research and Quality, 2) recommend a multimodal approach for bipolar disorder treatment involving pharmacotherapy, psychotherapy, and psychosocial interventions.

Emotion dysregulation (ED) plays a significant role in bipolar disorder (BD) and has gained considerable research interest. Higher ED is associated with more frequent mood episodes and poorer functionality in BD (3–5). Notably, these difficulties persist even during euthymic or remission phases (6, 7). Additionally, recent findings indicate that the combined effects of ED and alexithymia adversely impact quality of life and functionality, emphasizing the need to address these domains in therapy (8).

ER is a complex topic and there is no consensus on its definition. Some models emphasise the identification of strategies, such as Gross’s. An individual’s emotions and the way in which they are expressed are influenced by the type and timing of regulation strategies. Emotion regulation can occur at any of these stages of emotion generation i.e. at the level of both the antecedents of emotion and the emotional response (9). Emotional regulation strategies can be overt (i.e. behavioural) or covert (i.e. cognitive). Other approaches focus on emotional regulation skills (10), taking an ability-based view of whether or not a person can regulate his or her emotions. Emotional regulation corresponds to the typical or dispositional way individuals understand, consider and react to their emotional experiences (10). There are different abilities needed to regulate emotions, such as acceptance of emotions, engagement in goal-directed behaviours, control of impulsive behaviours, flexibility in using emotion regulation strategies perceived as effective, emotional awareness and emotional clarity (11, 12). Dysregulation corresponds to the disruption or deficit of one of these skills. Emotion regulation strategies and skills are, therefore, 2 different approaches, with bidirectional relationships between the two (10). As these two approaches are neither mutually exclusive nor in opposition, it is relevant to consider their integration in order to gain a more complete understanding of emotion regulation (13). More broadly, we need to continue developing work to better conceptualise emotion regulation and particularly within bipolar and related disorders.

Several studies have highlighted the potential role of ER difficulties across multiple mental disorders, underscoring its transdiagnostic nature (14). Given its association with mood dysregulation in the BD spectrum (15, 16), ER difficulties constitute a key target for psychological interventions focused on promoting coping with symptoms and personal recovery (as opposed to symptomatic remission) in this population

The key challenge remains to identify which treatments are effective for whom, as there is currently no established gold standard, and existing evidence presents a high risk of bias and an overall low quality. Understanding which approaches work best for specific patient populations is critical for advancing personalised care. Research endeavours aimed at identifying and developing more efficacious, readily accessible, and cost-effective psychotherapeutic interventions for BD are thus necessary.

As experts in mental health research and clinical practice, with a specialised focus on mood and emotion regulation in bipolar and related disorders, we identified a critical gap in practical guidance for effective interventions with this population. To address this, we established the European Network for Bipolar Emotion Regulation (ENBER), aiming to bridge the gap between empirical research and clinical practice. Our goal is to provide clinicians with actionable tools and insights that not only enhance patient care but also shape the direction of future research.

Our applied research on this topic is framed by the societies and healthcare systems in which we work. These share similarities but also have differences. Western European countries, in particular are often characterised by relatively high population density and levels of economic development, with implications for what can be delivered and what is considered affordable. The funding and governance of healthcare differs across European countries, including taxpayer-funded services with a relatively high degree of centralisation regarding decisions about provision and access (e.g. U.K. NHS, Portugal), and countries where funding is less centralised, deriving from a mix of taxation, user payment and health insurance schemes (e.g. France, Netherlands), where decisions about provision and access are often taken at a more local level and may reflect the judgements of private health insurers.

## Lessons learned from research

Several different frameworks have been used when investigating ER in BD, including Gross (17) Process Model and Response Styles Theory, which suggests specific strategies, and a clinical emotion dysregulation framework focused more on underlying deficits (11). Correspondingly, measures of ER developed for the general population that address overall difficulty with ER (Difficulties in Emotion Regulation Scale: 11) and use of multiple strategies (e.g. Cognitive Emotion Regulation Questionnaire: 18) are commonly used in research into BD. Also used are measures developed to capture use of bipolar-relevant ER strategies (most commonly the Rumination on Positive Affect scale: 19).

There are now several reviews and meta-analyses of research into ER and BD (15, 20, 21). At multiple levels (neural, behavioural, self-report), findings are not clear-cut. There is relatively compelling evidence that rumination (during negative affect) is significant for BD (15, 20). Less research has investigated strategies for regulating positive emotion (dampening, positive rumination) despite its relevance to BD (15). Findings suggest a pattern of maladaptive emotion regulation that is similar to that in other forms of psychopathology characterised by mood difficulties (20, 21).

Despite mixed findings, there is a tendency to suggest that emotion regulation is, therefore, a worthy target of psychological interventions for BD. The majority of research into emotion regulation in BD has explored the use of “adaptive” versus “maladaptive” strategies, however, the broader emotion regulation literature increasingly emphasises the importance of emotion regulation flexibility (10, 22), does the person have access to a repertoire of ER strategies, and are they able to select strategies appropriate to contextual demands? Why do people choose particular strategies? What might influence the impact of specific strategies on clinical and personal recovery outcomes? For example, positive rumination is related to increased symptoms but also to personal recovery (e.g., 23) therefore, under what circumstances is it adaptive or maladaptive? Future research in BD should consider not only which strategies are used but also the extent to which emotion regulation behaviours are flexible and context-appropriate. Given there is evidence that current mood states and individual differences variables influence the relationship between ER and bipolar symptoms (15), this research should also consider how current mood affects people’s capacity to make flexible and context-appropriate mood regulation choices.

To account for the role played by ER on the psychological well-being of people with BD, Koenders et al. (24) proposed a conceptual model including two broad profiles characterised by different strategies linked to approach and avoidance, each theoretically underpinned by different risk factors (e.g., temperament). The presence of distinct ER profiles in BD is yet to be tested, but adopting a person-centred methodology could be valuable in improving and personalising care. In other areas, approaches such as latent profile analysis have made some ground here and similar approaches could be used in BD to examine potential subgroups, including profiles seen in at-risk groups, given that ER is broadly considered a transdiagnostic risk factor for psychopathology.

Azevedo et al. (25) propose and discuss an adaptation of Linehan’s biosocial model for individuals with BD, that seeks to integrate the previous ideas (from 24) and further discuss pathways that lead to ED in people with BD. The biosocial model conceptualises emotional dysregulation as arising from the transaction between inherent biological characteristics (e.g., baseline emotional sensitivity, impulsivity) and social context, in particular an invalidating environment, which shapes and sustains emotional lability (26). While the biological vulnerabilities associated with BD are well-documented, the role of invalidating environmental factors remains underexplored in this population. However, with a growing body of evidence supporting DBT as a promising adjunctive treatment for BD, investigating early experiences and individuals’ recollections of invalidating responses to their emotions could shed light on the origins of emotion dysregulation in BD.

The transdiagnostic nature of ER is particularly pertinent to BD given the wide-ranging clinical phenomena experienced (depression, hypomania. mania, mixed states, commonly comorbid anxiety or ADHD, etc) and the non-specific nature of emerging symptoms outlined in staging models (27). We argue that there is a need to strike a balance between capitalising on transdiagnostic evidence (including for interventions) while also recognising the elements of ER that might be more specific to BD, including moderators/mediators of ER and differences in clinical and at-risk subgroups (familial risk, behavioural high-risk, subthreshold symptoms). Advanced research techniques, including real-time analysis of strategy combinations (e.g., through experience sampling), can help understand dynamic interactions between mood disturbances and ER in BD. This approach will hopefully allow for a better understanding of how emotional and mood states influence each other, offering valuable insights for intervention timing and strategy effectiveness. Yet, given the dynamic nature of emotions and mood, it is crucial to determine how they influence each other in people with BD.

This also means that not only are clinical intervention studies needed, but more experimental and controlled designs are required to study bipolar-specific mechanisms. In a recent systematic review (28), data was gathered on the methodological designs of experimental studies that aimed to test the leading psychological theories in BD (Reward Hypersensitivity Theory, Behavioural Activation System, Integrative Cognitive Model, Positive Emotion Persistence, Manic Defence and Mental Imagery). This review showed that various outcome variables and operationalisations were used across studies to test the same mechanism or theory. Additionally, mood induction and the use of physiological measures are underrepresented areas in the current designs. This suggests that there is a need for broader consensus on how specific psychological theories should be operationalised. Additionally, more standardised use of particular test batteries and questionnaires might increase comparability among studies and lead to a more systematic approach in bipolar disorder experimental research. Other methodological perspectives seem to be encouraged in view of improving the evaluation of ER in mental health, for example by developing more ecologically valid studies (29, 30), as well as evaluations in everyday life, which would enable flexibility (31) and the context of emotional regulation (29) to be taken into account. Finally, repertoire approaches to emotional regulation strategies are also a challenge for assessment (32). The challenge is to find scientific material that can capture the fact that an individual uses several emotion regulation strategies for the same emotional experience, which is termed emotional polyregulation (33).

## Lessons learned from clinical practice

Strategies focused on improving emotion regulation may form part of interventions such as Cognitive Behavioural Therapy (CBT) or may instead be the primary focus of interventions that teach skills in recognising and responding to intense emotional states, as well as managing the contexts and triggers that promote these, such as DBT. A recently adapted DBT Skills group intervention pilot study for people with BD provided interesting findings regarding emotion variance decrease across time. In this pilot RCT, the participants were assessed regarding their emotional intensity and mood (weekly across 12 weeks), and a significant decrease in their mood variance, as well as overall starting point, was observed (assessed only in the experimental group, through a diary card), particularly in sadness and anger intensity (results not published, see more in 25). Preliminary data analysed using linear mixed models with 3 time-points (pre, post and follow-up) and compared regarding the group they were attributed (experimental vs TAU) showed significant differences (favouring the intervention group) in their sense of personal recovery, quality of life and self-reassurance, and decreased self-criticism and difficulties in regulating emotions (25). In terms of the participants’ qualitative feedback on what was most helpful, the most recollected exercises, even at 3-month follow-up, included wise mind, opposite action and radical acceptance (as per the DBT skills training manual, see 34). Mindfulness exercises, in general, were also mentioned, and participants considered the ability to distance themselves and observe the train of thought particularly helpful when experiencing rapid thoughts. Some participants shared that they believed they could not do mindfulness when they were feeling like that because they could not stop their thoughts or even slow them down. It might be important to take this into consideration when working with these clients and teaching mindfulness exercises. When giving instructions, therapists might therefore encourage their person to observe rather than try to slow down their thoughts, as the former may feel more manageable. DBT skills training also seems feasible in a transdiagnostic context (including people with BD, ADHD and BPD) with no specific adaptations for people with BD. Indeed, regardless of the main diagnosis, results from a naturalistic study conducted in France suggest the continued use of DBT skills one year after the programme, improving the emotion dysregulation and social functioning of people with BD. This study suggests that transdiagnostic standalone DBT skills training groups may be useful for targeting ER (35).

Working with people with BD on emotion regulation raises the question of the functional overlap between the regulation of mood (hypomania and depression) and the regulation of emotion. To date, no research has directly examined whether difficulties in one area in people with BD are associated with difficulties in the other and the similarities and differences in the processes by which people regulate the two. This is particularly pertinent when considering therapeutic interventions for those individuals with BD who experience frequent mood swings, which may include cyclothymic and ultra-rapid cycling patterns. In a feasibility study of a DBT-informed approach for people with frequent bipolar mood swings, participants were offered face-to-face group skills training sessions and up to 8 brief individual coaching sessions (36). Additional content was included referring directly to (hypo)manic and depression in terms of recognition and regulation of these states. However, the original elements of the emotion regulation skills training module were retained and applied to both short-lived emotional responses and more sustained mood changes. Feedback from therapists and participants supported this approach: whilst specific reference to bipolar mood states was valued, emotion regulation skills were seen as applicable across a broad range of affective experiences, including high and low states.

Another understudied area is that of subtypes of BD – e.g., BD type 1 or 2, women with BD – and their specific needs in terms of ER interventions. Recently, Perrin & Weiner (in preparation)^1^ have delivered 12 individual sessions of DBT-informed psychotherapy to 4 women presenting with BD and premenstrual dysphoric disorder (PMDD). The authors found an improvement in ER and selfreported personal recovery following the intervention, suggesting the clinical pertinence of targeting ER in BD subgroups, which might be particularly affected by emotion dysregulation (37).

In addition to DBT and DBT-informed approaches, which target ER directly, interventions developed to tackle ER indirectly have also shown promising results. This is the case for instance of Mindfulness-Based Cognitive Therapy (MBCT), studies of which suggest improvements in ER for people with BD (38). Recently, using a multiple case study, Paulet and Weiner (39) reported that Imagery-Based Cognitive Therapy [ImCT; (40)] led to an improvement in emotion dysregulation in 5 participants out of 8, suggesting that the effects of imagery-based interventions on ER should be further investigated. Importantly, these studies point to potential mechanisms involved in ER difficulties in people with BD (e.g., lack of emotional awareness, imagery disturbances), which might be worthy additions to existing psychological treatments.

## Discussion and future directions

This perspective synthesises insights and recommendations derived from the clinical and research efforts of the European Network for Bipolar Emotion Regulation (ENBER). By sharing lessons learned from clinical practice and research with individuals with bipolar disorder (BD), this discussion aims to highlight key questions that warrant further investigation given the current state of knowledge on ER in BD.

In light of these reflections, we would argue there is value in continuing to explore the potential of ER focused interventions for people with BD. In particular, it will be important to explore whether incorporating ER strategies into treatment plans not only aids with symptom management but also equips patients with a toolkit of strategies to navigate various emotional and mood states effectively. Our clinical experience is that supporting patients in developing a repertoire of ER strategies to select appropriate strategies based on contextual demands can, for some, significantly enhance their ability to manage the wide range of emotional and mood states characteristic of BD.

Preliminary studies and qualitative feedback have shown promising acceptance of adapted Dialectical Behavioural Therapy (DBT) skills for individuals with BD. Skills such as mindfulness, wise mind, opposite action, and radical acceptance were identified by participants as useful in mood and emotional regulation. However, further studies are needed to refine these adaptations and understand what is needed in which order, and to explore mechanisms of change.

Another consideration is that of customising interventions for specific subgroups of individuals with BD. Personalising treatment for BD involves addressing individual variations in symptomatology, ER abilities, and genetic profiles. This multidisciplinary approach combines psychiatric and psychological interventions, epigenetics, and advanced mood-monitoring tools. Personalising treatment to the individual's needs has been identified as a way to optimise patient outcomes, reduce symptom severity, and improve overall quality of life. Future directions include refining genetic and neurobiological research to predict treatment responses, assessing psychotherapeutic approaches to identify effective strategies for different subgroups, and enhancing the accessibility of individualised and personalised care. Future research in BD should consider not only which strategies are used but also the extent to which emotion regulation behaviours are flexible and context appropriate. **Table 1** summarises our views on key topics for future research that need further development.

**Table 1:** ENBER Summary of Key Research Directions in emotion regulation (ER) and bipolar disorder (BD).

Research area	Description
Treatment improvements and mechanisms	
ER Profiles Related to BD and personalised approaches	Identify ER profiles that increase susceptibility to a more severe BD course, examining which patterns of ER increase vulnerability to worsened symptoms and mood instability.
Treatment Optimisation for ED in BD	Conduct rigorous studies to determine the most effective treatment methods for addressing ED in the context of BD.
Optimising ER treatment	Further exploration of ER psychological intervention for BD, focusing on refining and optimising treatment strategies. This includes identifying which elements of ER psychoeducation and training have the most significant impact on treatment outcomes when included or excluded in treatment protocols and in which sequence/order.
Aetiology and Symptoms: Connection with ER	
Contextual Adaptiveness of ER Strategies	Investigate the contexts in which specific ER strategies may be adaptive vs. maladaptive for people with BD, considering the impact of mood states on the effectiveness of ER strategies.
Aetiology of Emotional Dysregulation (ED) in the context of the biosocial model	Investigate ED in BD through the biosocial model to understand its development (i.e. elements of invalidating environment and biology/epigenetic) and how it interacts with other aspects of BD pathology.
Overlap Between Symptom and Emotion Regulation	Study the overlap between mood regulation, symptom regulation, emotion regulation, mood instability, and emotion instability to clarify these relationships and identify specific ER challenges in BD.
Methodology	
Measurement Development for ER Repertoire	Develop and refine measurement tools for assessing the ER repertoire in BD, with an emphasis on both flexibility and adaptability in different contexts.
Experience Sampling Methodology (ESM)	Increase the use of ESM to capture real-time data on ER, mood states, and BD symptomatology, allowing for more nuanced insights into adaptive vs. maladaptive ER in various contexts.
Consensus on Measurement	Achieve consensus on standardized measurement tools for ER in BD to facilitate cross-study comparisons and improve research reliability.
Qualitative Approaches to ER Phenomenology	Employ qualitative methods to explore the lived experiences of ER difficulties among people with BD, providing a richer, more nuanced understanding of the challenges faced in daily life.
Recovery and Service users’ perspective	
Listen to service users/expert by experience	Qualitative research and collaborative projects need to be done, including service users from the start (i.e., co-deseing) to improve the understanding of what counts as valued outcomes and as recovery in the journey of people with BD.

This perspective paper seeks to bring together the expertise of different researchers and clinicians from different countries around Europe with shared research and clinical interests (the ENBER network) and thus approach ER in bipolar disorder with a multidisciplinary and multicultural perspective. Our reflections underscore the importance of better and sound methodologies to capture mood variance and to contribute to a better understanding of the emotional experiences of people with BD, as well as the need to further investigate the mechanisms and strategies that underpin them. Additionally, incorporating patients as co-designers in the research process bridges the gap between theoretical frameworks and the experience of living with BD, enriching our understanding of what constitutes meaningful recovery and how to access it. Together, these insights can pave the way for more effective, personalised interventions with the potential to improve the overall care and quality of life received by individuals with bipolar disorder.

## Footnotes

1 Perrin L, Weiner L. (in preparation). Treating emotion dysregulation in women with premenstrual dysphoric disorder and bipolar disorder using DBT.
